# Analysis of genetic diversity of Tunisian caprifig (*Ficus carica* L.) accessions using simple sequence repeat (SSR) markers

**DOI:** 10.1186/s41065-015-0002-9

**Published:** 2015-10-22

**Authors:** Awatef Essid, Fateh Aljane, Ali Ferchichi, Jose Ignacio Hormaza

**Affiliations:** 1 Laboratoire d’Aridoculture et Cultures Oasiennes Institut des Régions Arides de Médenine, Médenine, 4119 Tunisia; 2 Institut National Agronomique de Tunisie, 43 Avenue Charles Nicolle, 1082 Cité Mahrajène, Tunis, Tunisia; 3 Subtropical Fruit Crops Department, Instituto de Hortofruticultura Subtropical y Mediterránea la Mayora’, (IHSM-CSIC-UMA), 29750 Algarrobo-Costa, Málaga, Spain

**Keywords:** Caprifig, *Ficus carica*, Fig, Microsatellites, Moraceae, SSRs, Tunisia

## Abstract

**Background:**

The common fig (*Ficus carica* L.) is a gynodioecious species with two sexual forms: male trees (caprifigs) with male and female flowers and female trees that produce only female flowers that will result in the edible fig syconium. In this study the genetic diversity of 20 Tunisian accessions of caprifig is analyzed using SSR markers previously developed for this crop.

**Results:**

The results revealed that the 13 pairs of primers used amplified a total of 37 alleles in the accessions studied. The number of alleles per locus ranged from two to six, with a mean value of 2.85 alleles per locus. Observed and expected heterozygosities showed mean values of 0.33 and 0.29 respectively. UPGMA cluster analysis and Principal Component Analysis grouped the caprifig accessions analyzed in three groups.

**Conclusion:**

The results obtained show a low genetic diversity in the Tunisian accessions of caprifig studied and, in spite of analyzing samples from different geographic regions, no clear groupings based on geographical origin are observed suggesting widespread exchange of caprifig plant material through vegetative propagation among different areas in Tunisia.

## Background

The common fig (*Ficus carica* L.; 2n = 2x = 26) [1] belongs to the Moraceae, a family with over 1400 species distributed in about 40 genera. The genus *Ficus* L. contains about 750 species of woody trees, epiphytes and shrubs, mainly of tropical and subtropical distribution, divided into six subgenera [2, 3] that share a unique inflorescence, the syconium. Common fig seems to be originated from Southern Arabia and the eastern part of the Mediterranean regions and, together with the grapevine and the olive, is considered one of the three classical fruit trees associated with the beginning of horticulture in the Mediterranean Basin [4–6] domesticated at a very early stage contemporarily with cereal crops [7].

*Ficus carica* L. is a gynodioecious species with two sexual forms: male trees (caprifigs) that produce syconia with separate male and female flowers and female trees that produce syconia with only female flowers that will develop into edible seeded figs if pollinated. Since only male trees produce pollen the common fig is functionally a dioecious species. Three types of female figs are cultivated [8]: the common-type that develops fruit parthenocarpically without pollination and can produce one (unifera varieties) or two (bifera varieties) crops, the Smyrna-type that requires pollination with pollen from caprifigs, and the San Pedro-type that produces a first crop parthenocarpically (breba) and a second crop (fig) only after pollination with pollen from caprifigs. Pollination in the genus *Ficus* is dependent on the coevolution of *Ficus* species with pollinating wasps of the family Agaonidae [9]. In the case of the common fig, pollination (caprification) is performed by a specific pollinating insect, *Blastophaga psenes* L. The caprifigs produce fruits in three crop cycles during each growing season, each harboring the larvae, pupae and, temporarily, the adults of the pollinating *Blastophaga* [8]: “profichi” that ripen in early summer, “mammoni” that ripen in autumn and “mamme” that overwinter on the tree and mature in spring. Only the "profichi” carry pollen and are used for pollination. Pollination occurs naturally when female and caprifig trees are present in the same orchard or when caprifig branches with flowers are placed close to female fig trees. The main problems of caprification in Tunisia, which are common to other fig producing areas, are the disruption of the cycle of *Blastophaga psenes* in cold mountainous areas and the unavailability of mature caprifig “profichi” when female figs are receptive.

In Tunisia, as in other Mediterranean countries [10], fig has been traditionally cultivated since ancient times in diverse edaphoclimatic conditions, in association with date palms in the south or olive trees in other regions of the country resulting in a high number of local varieties and frequent exchange of varieties among different regions [11, 12]. The denominations of the cultivars are usually based on the color, size and time of fruit ripening or geographical origin resulting in confusion in nomenclature. Hence, appropriate characterization and differentiation among cultivars is necessary to optimize fig germplasm management and conservation, jeopardized by intensive urbanization, cultivation of selected clonal varieties or biotic and abiotic stresses. This genetic erosion is even more important in caprifigs since they do not produce edible fruits and, consequently, the pressure to conserve genetic resources is lower. However, artificial caprification is still a common practice in Tunisian fig cultivation and, consequently, caprifigs are used by farmers in order to obtain edible fig production [12].

Various studies have reported the use of morphological traits [11–19] and isozyme markers [20, 21] for fig characterization. However, these parameters are influenced by environmental conditions and the phenological status of the trees. To overcome these difficulties, different molecular tools such as RAPDs [13, 20–27] ISSRs [6, 19, 24, 26–28], AFLPs [20, 29, 30], RFLPs [6] or SSRs [6, 10, 26, 27, 30–41] have been used for fig germplasm characterization and diversity analyses. However, most of those studies include mainly female fig cultivars and molecular characterization and diversity studies in caprifigs are very scarce.

The main objective of this study was to characterize and evaluate the genetic diversity of 20 Tunisian caprifig accessions using SSR markers in order to develop strategies to preserve the endangered genetic resources of this species.

## Methods

### Plant material

**Fig. 1:**
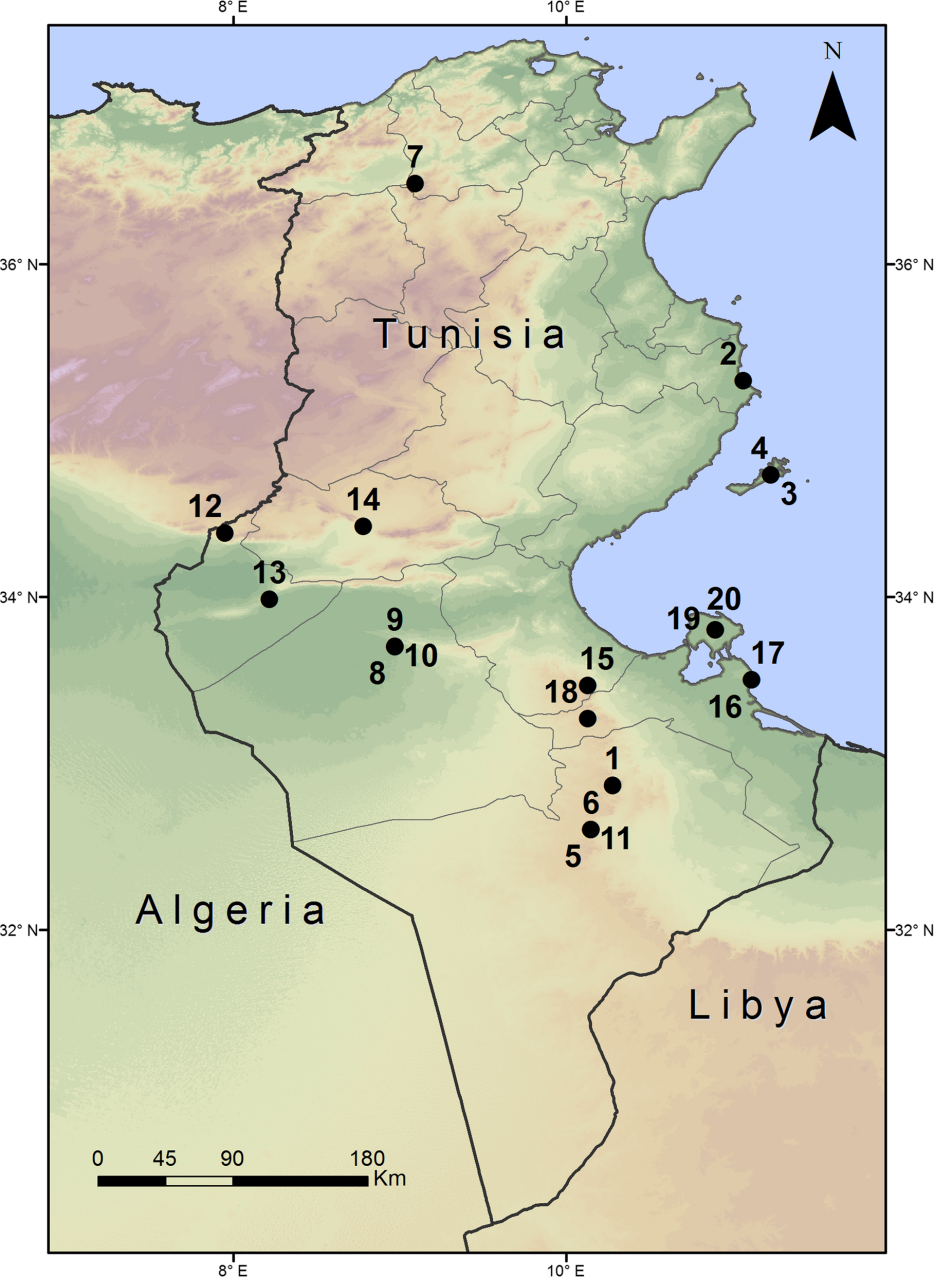
Map showing the geographic localization of the different Tunisian caprifig accessions studied in this work

This study was carried out on 20 Tunisian caprifig accessions originated in different geographic regions and with different phenotypic traits (Table 1 and Fig. 1). All the accessions were planted in the fig germplasm collection of the Arid Land Institute of Médenine established in El Gordhab, Tataouine in Southeastern Tunisia. The plant material was propagated by hardwood cuttings.

**Table 1:** Names and localities of origin of the 20 Tunisian caprifig accessions studied in this work

No.	Accession name	Locality of origin (Governorate)	Syconia shape	External syconia color	Internal syconia color	Number of leaf lobes
1	Magouli1	Douiret (Tataouine)	Globose	Light green	Light pink	Three
2	Jrani	Ghadhabna (Mahdia)	Globose	Purple green	Light yellow	Three
3	Bithri1	Kerkennah (Sfax)	Oblong	Purple green	Light yellow	Five
4	Assafri	Kerkennah (Sfax)	Oblong	Purple green	Light yellow	Three
5	Bouharrag1	Bir Amir (Tataouine)	Globose	Light green	Dark pink	Five
6	Bithri2	Bir Amir (Tataouine)	Oblong	Light green	Light pink	Three
7	Dhokkar1	Djebba (Béja)	Oblong	Light green	Light yellow	Three
8	Limi	Kébéli (Kébéli	Oblong	Green	Dark pink	Three
9	Tebessi	Kébéli (Kébéli)	Oblong	Green	Dark pink	Five
10	Sawoudi	Kébéli (Kébéli)	Oblong	Purple green	Light pink	Five
11	Magouli2	Bir Amir (Tataouine)	Oblong	Light green	Light pink	Five
12	Dhokkar2	Tamaghza (Tozeur)	Oblong	Purple green	Light yellow	One
13	Dhokkar3	Dégâche (Tozeur)	Oblong	Purple green	Light yellow	Five
14	Dhokkar4	Gafsa	Oblong	Dark purple	Light yellow	Three
15	Bouharrag2	Toujen (Gabés)	Globose	Light green	Dark pink	One
16	Beldi	Zarzis (Médenine)	Oblong	Purple green	Light yellow	Five
17	Dhokkar6	Zarzis (Médenine)	Oblong	Purple green	Light pink	Three
18	Dhokkar7	Zammour (Médenine)	Oblong	Purple green	Light yellow	Three
19	Bouharrag3	Djerba (Médenine)	Globose	Dark green	Dark pink	Five
20	Khadhouri	Djerba (Médenine)	Globose	Green	Dark pink	Three

### Genomic DNA extraction and PCR amplification

Genomic DNA was extracted from lyophilized young leaves following a CTAB-based method optimized previously for fig [33]. After purification, DNA quantity and quality were checked using a Nanodrop ND-1000 UV-visible spectrophotometer, diluted to 10 ng μl^−1^ with modified TE buffer (1 M Tris–HCl pH 8.0; 0.5 M EDTA) and used for PCR amplification.

A set of 13 SSR loci (MFC1, MFC2, MFAC4, LMFC15, LMFC18, LMFC21, LMFC23, LMFC24, LMFC27, LMFC28, LMFC30, LMFC31 and LMFC32), previously developed in fig [31, 33] were used in 15 μl reactions containing 16 mM (NH4)2SO4, 67 mM Tris–HCl, pH 8.8, 0.01 % Tween 20, 2 mM MgCl2, 0.1 mM each dNTP, 0.4 μM each primer, 25 ng genomic DNA and 0.5 Units of BioTaq DNA polymerase (Bioline, London, UK). Amplifications were performed in a thermocycler (Bio-Rad Laboratories, Hercules, CA, USA) using the following temperature profile: an initial step of 1 min at 94 °C, 35 cycles of 30 s at 94 °C, 30 s at 55 °C and 1 min at 72 °C, and a final step of 5 min at 72 °C. The amplification products were resolved using a CEQ^TM^ 2000XL capillary DNA analysis system (Beckman Coulter, Fullerton, CA, USA).

### Data analysis

Different parameters of genetic diversity were estimated: number of alleles per locus (A), allelic frequencies, observed heterozygosity (Ho), expected heterozygosity (He), Wright’s fixation index and probability of identity (PI). Those parameters were only computed using the SSRs that produced amplification of a single locus (e.g. one or two alleles amplified in each of the samples analyzed). The computations were performed with the program POPGENE 1.32 [42] and IDENTITY 1.0 [43]. Genetic relationships within the accessions studied were calculated using UPGMA cluster analysis of the similarity matrix obtained from the proportion of shared amplification fragments [44] using the program NTSYS 2.11 (Exeter Software, Stauket, NY). The cophenetic correlation coefficient was estimated by comparing with the Mantel test the cophenetic matrix obtained from the dendrogram with the original similarity matrix. The bootstrap values were obtained using 2000 replicates with TREECON 1.3b [45]. Principal Component Analysis (PCA) was performed using NTSYS 2.11(Exeter Software, Stauket, NY).

## Results and discussion

### Genetic polymorphism and SSR patterns

The 13 SSR loci produced successful and repeatable amplification fragments in all the 20 caprifig accessions analyzed, resulting in a total of 37 alleles ranging from two (LMFC32, LMFC15, LMFC21, LMFC31, LMFC18, LMFC27 and LMFC23) to six (LMFC30) alleles per locus (Table 2), with an average of 2.85 alleles per locus and amplification fragment sizes between 120 and 278 bp. Some accessions showed the amplification of more than two alleles (three alleles in LMFC30, MFC1, MFC4 and four alleles in LMFC28) suggesting the probable amplification of more than one locus. Similar results for those 4 loci were obtained by Giraldo et al. [35] analyzing an ex situ germplasm collection of 209 fig accessions. The remaining 9 SSRs showed one or two bands per genotype, suggesting the amplification of a single locus. For these 9 loci, the accessions studied were considered homozygous or heterozygous when one or two fragments were present per locus respectively [46]. Genetic diversity was studied with the 9 loci that produced one or two alleles per locus. For these loci, allelic frequencies varied from 0.03 to 0.98 with a mean of 0.45 (data not shown). Furthermore, 15 % of the alleles studied were considered rare (p < 0.05) and fixed (p > 0.9). Additionally, six alleles were found only in one accession (allele 205 of LMFC32 is present only in ‘Jrani’; allele 278 of LMFC24 is present only in ‘Dhokkar2’; allele 271 of LMFC21 is present only in ‘Dhokkar3’; allele 256 of LMFC30 is present only in ‘Dhokkar1’ and allele 193 of LMFC 28 is present in ‘Bouharrag2’).

**Table 2:** Locus name, range size, allele number (A), observed (Ho) and expected (He) heterozygosity, probability of identity (PI) and fixation index calculated for 20 Tunisian caprifig accessions

Locus	Size (bp)	A	Ho	He	PI	F
LMFC15	205–207	2	0.50	0.42	0.60	−0.19
LMFC18	120–126	2	0.25	0.22	0.68	−0.14
LMFC21	265–272	2	0.05	0.05	0.91	−0.03
LMFC23	132–134	2	0.10	0.10	0.83	−0.05
LMFC24	274–278	3	0.25	0.45	0.55	0.45
LMFC27	186–196	2	0.55	0.40	0.60	−0.38
LMFC28	183–200	5				
LMFC30	231–258	6				
LMFC31	228–242	2	0.80	0.50	0.62	−0.62
LMFC32	205–209	2	0.05	0.05	0.91	−0.03
MFC1	176–192	3				
MFC2	157–170	3	0.40	0.43	0.45	0.08
MFC4	198–221	3				
Mean			0.33	0.29	0.68	−0.10

Observed heterozygosity ranged from 0.05 (LMFC32 and LMFC21) to 0.80 (LMFC31) with a mean of 0.33 (Table 2). The expected heterozygosity ranged from 0.05 (LMFC32 and LMFC21) to 0.50 (LMFC31) with a mean of 0.29 (Table 2). A heterozygote excess (H_obs_ > H_exp_) was observed for the LMFC15**,** LMFC31, LMFC18, LMFC27 loci whereas a deficit of heterozygosity (H_obs_ < H_exp_) was observed in LMFC24 and MFC2 loci (Table 2). For the loci LMFC 32, LMFC21 and LMFC23 observed and expected heterozygosities were similar (Table 2). The maximum value of the probability of identity (0.91) was detected in LMFC32 and LMFC21 and the minimum (0.45) in MFC2 (Table 2).

The diversity parameters obtained in this work are mostly below the range of those reported for fig using microsatellites in previous works that mainly analyzed female cultivars [6, 10, 26, 27, 30, 41]. Although most of those works included the analysis of a higher number of female genotypes from different origins, the results obtained suggest a low genetic diversity in the Tunisian caprifig accessions studied (see below).

## Cluster and principal component analyses

**Fig. 2:**
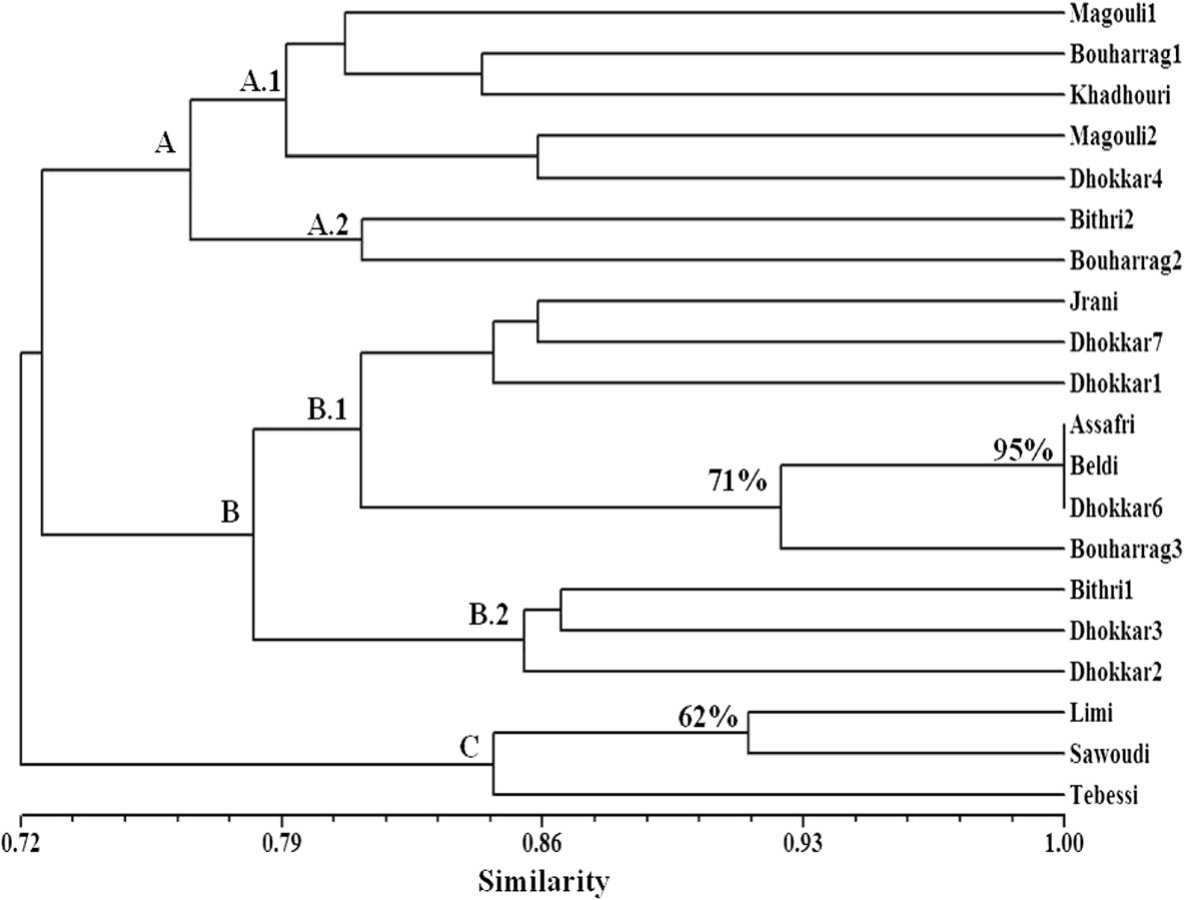
UPGMA dendrogram of 20 Tunisian caprifig accessions based on SSR markers. Bootstrap values are shown if 50 % of higher. Capital letters represent assigned clusters

Among all the possible UPGMA dendrograms generated, that with the highest cophenetic correlation (*r* = 0.76) between the cophenetic coefficient and the similarity matrix was chosen. The UPGMA dendrogram obtained showed three main groups among the genotypes analyzed (Fig. 2) with three undistinguishable accessions that represent a case of synonymy (Assafri from Kerkennah, Beldi and Dhokkar6 from Zarzis). The first group (**A**) includes 7 accessions (Magouli1, Bouharrag1, Khadhouri, Magouli2, Dhokkar4, Bithri2 and Bouharrag2), the second (**B**) contains 10 accessions (Jrani, Dhokkar7, Dhokkar1, Assafri, Beldi, Dhokkar6, Bouharrag3, Bithri1, Dhokkar3 and Dhokkar2) and the third (**C**) includes 3 accessions (Limi, Sawoudi and Tebessi). Several homonymies were detected in the genotypes analyzed since genotypes having the same name in the same or different locations are genetically different. This includes all the different samples analyzed of Magouli (2 samples), Bouharrag (3 samples), Bithri (2 samples) and Dhokkar (6 samples). Among all the nodes obtained only three groups have bootstrap values higher than 50 %. These groups include the three undistinguishable accessions (Assafri, Beldi and Dhokkar6) (bootstrap of 95 %), those three accessions and Bouharrag3 (bootstrap of 71 %) and the accessions Limi and Sawoudi (bootstrap of 62 %).

**Fig. 3:**
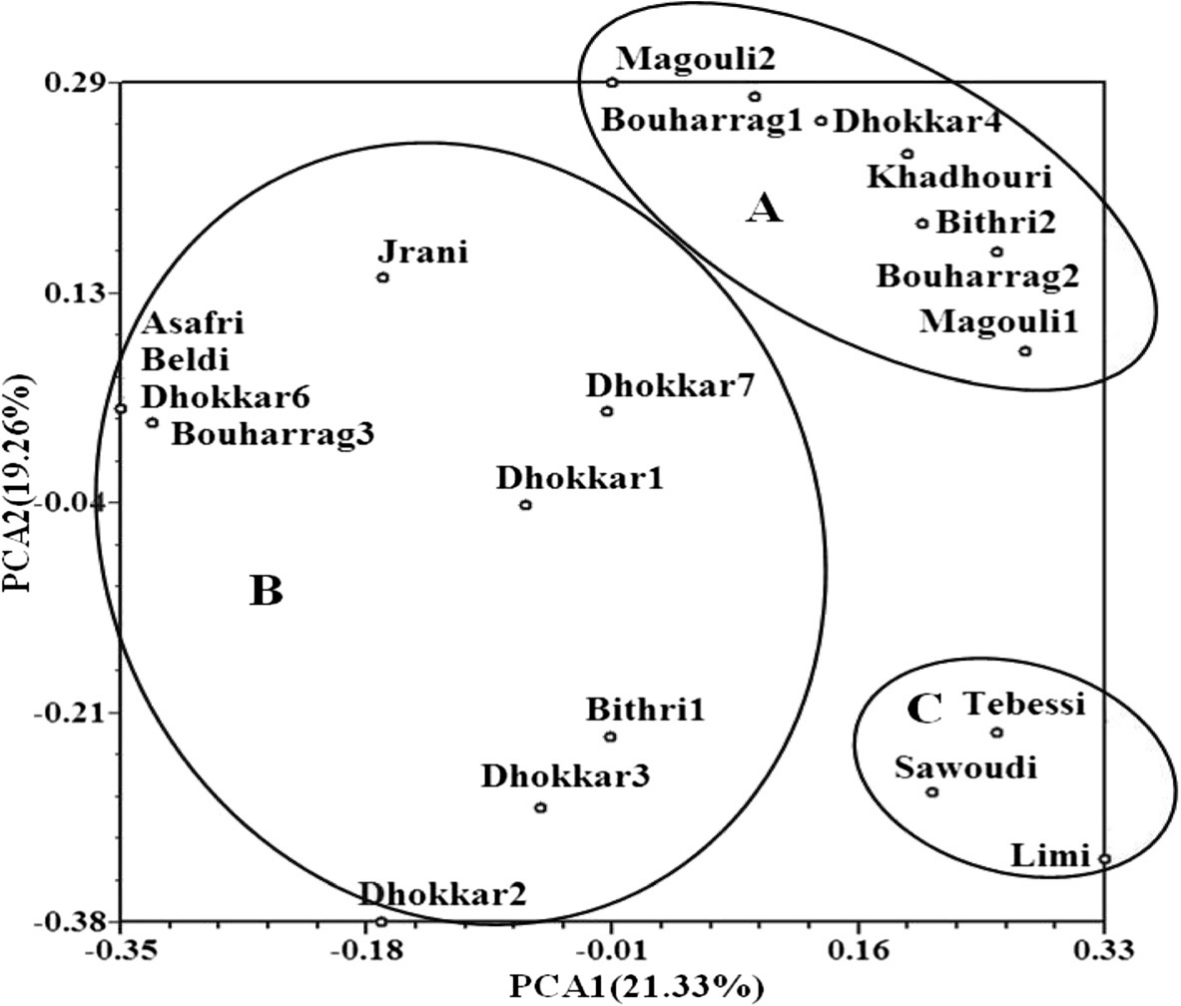
Principal component analysis grouping 20 Tunisian caprifig accessions based on SSR markers

Principal Component Analysis shows that the three first principal components explain 50.50 % of the total variability. The contributions of PCA1, PCA2 and PCA3 were 21.33, 19.26 and 9.92 % respectively. Fig. 3 shows the distribution of accessions according to the first two components (PCA1 and PCA 2) in which three groups that correspond to those found in the UPGMA analysis can be clearly identified.

Some of the main groups obtained in the UPGMA and PCA studies are correlated with the geographic origin of the genotypes. Thus the group C in Figs. 2 and 3 corresponds to three cultivars (Limi, Tebessi and Sawoudi) from the same location in Southwestern Tunisia (Kébéli) and the group of three undistinguishable cultivars (Assafri, Beldi and Dhokkar6), which are probable synonymies, were originated in Eastern Tunisia. Six of the accessions, even from different geographic origins, wear the name ‘Dhokkar’; although this could be a case of homonymy, it has to be considered that ‘dhokkar’ is the common word for caprifig in Arabic. Regarding the rest of the accessions analyzed, although some of the ones that cluster together (such as Dhokkar2 and Dhokkar3) have a common geographical origin, no clear geographical groupings can be found for most of them. Similar results have also been obtained with common type figs in Tunisia using different types of molecular markers [24, 28] These results could suggest a common genetic base for most Tunisian caprifigs that can be explained by the easy vegetative propagation of the crop that allows exchange of plant material between different regions. This contrasts with a higher genetic diversity found in Tunisian common figs by characterizing cultivated and wild figs [41].

## Conclusion

The present study provides the first molecular database of Tunisian caprifig accessions using SSR markers. The set of SSR markers used indicates that the genetic diversity between accessions studied is overall narrow and that no clear relationship is found between geographical origin and genetic composition suggesting exchange of caprifig genetic material among different regions. Additional studies with caprifigs from other countries should be performed in order to have a clear picture on overall caprifig genetic diversity and optimize collaborative caprifig genetic resource management and conservation.
